# Prevalence of Overweight and Obesity in Children of Immigrant Origin in Spain: A Cross-Sectional Study

**DOI:** 10.3390/ijerph19031711

**Published:** 2022-02-02

**Authors:** Joaquín Moncho, Alba Martínez-García, Eva Mª Trescastro-López

**Affiliations:** 1 Research Unit for the Analysis of Mortality and Health Statistics, Department of Community Nursing, Preventive Medicine, Public Health and History of Science, University of Alicante, 03080 Alicante, Spain; joaquin.moncho@ua.es; 2 Balmis Research Group in History of Science, Health Care and Food, University of Alicante, 03080 Alicante, Spain; eva.trescastro@ua.es

**Keywords:** immigrants, childhood obesity, diet, nutritional health behavior, overweight, obesity, socioeconomic factors, health, inequality

## Abstract

Social determinants of health are a major contributing factor to health inequalities and have important effects on the health of the population. The aim of the study was to estimate the prevalence of overweight/obesity in native and immigrant children aged 2–14 years residing in Spain, and to explore its relationship with socioeconomic factors, habits, and living and health conditions. This study used data from the 2017 Spanish National Health Survey, which collects health information on the population residing in Spain. Multivariate logistic regression models were fitted to estimate the risk of overweight/obesity controlling for the variables of interest. The final sample consisted of 2351 households. Children of immigrant origin had a significantly higher overall prevalence of overweight and/or obesity than natives, both in boys (40.5% vs. 29.5%) and girls (44.8% vs. 30.3%), and a higher adjusted risk of overweight/obesity (OR = 1.67). In addition, it has been observed that children of immigrant origin were at higher risk of consuming sugary soft drinks, exercising less, and using screens more. In conclusion, the increased risk of having different habits that may contribute to developing excess weight and of having overweight/obesity in children with a migrant background should guide public health policies and interventions, emphasizing those groups at higher risk and incorporating considerations of socioeconomic inequalities.

## 1. Introduction

Social determinants of health, such as socioeconomic, cultural, and/or environmental conditions, are major contributing factors for health inequalities and have important effects on population health [1,2,3,4]. Socioeconomic factors, such as income level, education, sex/gender, or ethnicity, have a direct association with diet [5] and excess weight [6]. Childhood obesity is associated with an increased risk of many harmful comorbidities in adulthood, such as diabetes, hypertension, hypercholesterolemia, and/or cardiovascular disease [7,8].

Overweight and obesity figures have increased over the last few decades, both in children and adults, worldwide and in Spain [9,10,11]. According to the World Health Organization (WHO) report, European Childhood Obesity Surveillance (COSI), Spain ranks first in terms of the prevalence of childhood obesity in Europe, with 17.7% [12].

Likewise, in Spain, according to data from the National Institute of Statistics (INE) for the year 2020, foreign residents make up 11.25% of the population, with an increase in recent years [13]. This population group is at greater risk of suffering health inequalities due to the socioeconomic factors that affect their lives [1]. In addition, food insecurity is a common problem among migrants compared to non-migrants, and is determined by cultural and religious beliefs, and sociodemographic, economic, and environmental factors, as well as education and lifestyle changes [14]. These components have been associated with a number of negative health and behavioral indicators, such as weight and lifestyle factors, particularly in the child population [14]. Several systematic reviews [14,15] indicate possible associations between migrant populations and the increased likelihood of having overweight and obesity. For these reasons, and due to the increase in the immigrant population in Spain, it is important to identify and address the social determinants of health, and the dietary and lifestyle habits that lead to health disparities in this group.

The aim of this study is to estimate the prevalence of overweight/obesity (OW/OB) in native and immigrant children aged 2–14 years living in Spain and to explore its possible relationship with socioeconomic factors, habits, and living and health conditions.

## 2. Materials and Methods

### 2.1. Data Source

This study used data from the 2017 Spanish National Health Survey [11] conducted by the Ministry of Health, Consumer Affairs and Social Welfare in collaboration with the National Institute of Statistics, which collects health information on the population residing in Spain and consists of 3 questionnaires: household, adult, and child. The questionnaires were self-reported. In the case of minors, the questionnaires were completed by parents or legal guardians This study initially considered households in which the selected adult was the parent of the selected child and the age of the selected child was between 2 and 14 years. Under these criteria, 4129 households were selected. Finally, to carry out this study, households were selected in which the adult selected was the mother of the minor; this resulted in a final database of 2351 households.

### 2.2. Variables

#### 2.2.1. Dependent Variables

The dependent variables were: overweight and/or obesity determined from the weight, height, age, and sex of the children using the International Obesity Task Force (IOTF) definition [16]. The frequency of physical exercise outside school was classified into two categories: (1) no-some exercise: no exercise or free time is spent almost completely sedentary in activities such as reading, watching TV, going to the cinema, etc., the use of mobile phones, tablets, TV, etc., or does some occasional physical or sport activity, such as walking or cycling, gentle gymnastics, or recreational activities requiring light exertion, etc.; (2) frequent or intensive: does physical activity several times a month, such as sports, gymnastics, running, swimming, cycling, team games, etc., or does sports or physical training several times a week. The frequency of consumption of certain foods were examined, including fresh fruit (excluding juices), meat, eggs, fish, pasta–rice–potatoes, bread–cereals, vegetables–salads, pulses, sausages–cold meat, dairy products (milk, cheese, and yoghurt), sweets (biscuits, pastries, sweets, jams, marmalades, etc.), soft drinks with sugar, food with sugar, etc., fast food (fried chicken, sandwiches, pizzas, or hamburgers), and snacks or savory finger foods (chips, croissants, or pickles).

Frequencies of consumption were initially grouped into three categories (less than once a week, between one and three times a week, and more than three times a week). On the other hand, another grouping of foods (healthy/unhealthy) was used, following the criteria of the Dietary Recommendations for the Spanish Population set out in the Report of the Scientific Committee of the Spanish Agency for Food Safety and Nutrition (AESAN) [17]. According to these recommendations, the consumption of sweets, soft drinks with sugar, fast food, and snacks one or more times a week was classified as unhealthy. Similarly, no or occasional physical exercise and screen-use more than one hour per day on weekdays or weekends were classified as unhealthy, according to the WHO guidelines on physical activity, sedentary behavior, and sleep for children under 5 years [18], and the WHO physical activity recommendations for the population aged 5–17 years [19].

#### 2.2.2. Explanatory Variables

The main explanatory variables of interest were: origin of the child (country of birth of the mother: Spain vs. others), sex and age of the child (age in years was considered as a continuous quantitative variable and categorized into 3 age groups: 2–5, 6–9, and 10–14 years), educational level of the selected adult (classified into three categories: University, Higher Vocational Training, and Basic–Intermediate), perception of the selected adult’s weight of the child (it was classified into two categories: normal or below normal, and fairly or somewhat above normal), overweight and/or obesity of the adult, type of cohabitation (couple or other), and social class based on the occupation of the reference person in the household. The social class categories considered were based on the classification proposed in 2012 by the Working Group on Determinants of the Spanish Society of Epidemiology (SEE), adapted for the ENSE [20]. These are obtained from the occupation, current or past, and coded to three digits according to the National Classification of Occupations that came into force in 2011 (CNO-11) [21]. The 6 categories proposed were: I. directors and managers of establishments with 10 or more employees and professionals traditionally associated with university degrees; II. directors and managers of establishments with less than 10 employees, professionals traditionally associated with university degrees, and other technical support professionals. Sportsmen and artists; III. intermediate occupations and self-employed workers; IV. supervisors and workers in skilled technical occupations; V. skilled primary sector workers and other semi-skilled workers; and VI. unskilled workers. For the analysis, these 6 categories were regrouped into 3 categories as follows: (1) Class I–II; (2) Class III–IV; and (3) Class V–VI.

### 2.3. Data Analysis

Means, standard deviations, and 95% confidence intervals were calculated for BMI according to sex, age group, and origin of the child. Student’s *t*-test was used to compare BMI by origin of the child. To compare the prevalence of overweight and/or obesity, or the frequency of food consumption at the levels defined by the explanatory variables, the chi-square test or Fisher’s exact test was used, when necessary, as well as the Gamma measure of association. Finally, multivariate logistic regression models were constructed to adjust the effects of the different explanatory variables of interest, and the corresponding adjusted odds ratios were obtained by taking into account in all cases the possible interaction and collinearity effects. The analyses were performed with the statistical analysis program SPSS 26.0 (IBM, Armonk, NY, USA). In all analyses, the data were weighted due to the complex sample design.

## 3. Results

The final sample consisted of 2351 households in which the selected adult was the mother of the child. Table 1 shows the average body mass index of children of native and immigrant origin, disaggregated by age and sex. Overall, children of immigrant origin had a significantly higher average BMI than native children in both sexes (18.30 vs. 19.67 in boys, *p* = 0.003; 17.94 vs. 19.29 in girls, *p* < 0.001). By age group, statistically significant differences were observed in girls, in all cases, with higher body mass indices in children of immigrant origin. The largest differences in girls were detected in the age groups 6–9 (18.71 vs. 17.23) and 10–14 (21.11 vs. 19.37). In boys, it was also higher in those of immigrant origin, but the differences were not significant. When analyzed considering age year by year, children of immigrant origin had higher BMIs than those of native origin at practically all ages, both in boys and girls.

Table 2 shows the prevalence of OW/OB overall and according to certain socio-demographic characteristics disaggregated by the sex and origin of the child. The overall prevalence of OW/OB in all variables was significantly higher in children of immigrant origin, both in boys (40.5% vs. 29.5%, *p* = 0.004) and girls (44.8% vs. 30.3%, *p* < 0.001).

Children of native origin had a lower prevalence of OW/OB when the mother had university studies (24.4% and 30.2% in boys and girls respectively vs. 51.1% and 31.5% for basic–intermediate studies), although it was only significant in boys. For the same level of education of the mother, children of immigrant origin showed higher prevalence of OW/OB in all cases except in children whose mother had basic–intermediate studies, where it was higher in natives, although not significant. These differences were significant in the case of university studies (in boys and girls) and in girls whose mothers had basic–intermediate studies.

Overall, a higher prevalence of overweight and OW/OB was also observed in children whose mother did not live with a partner. The greatest difference was detected in children of immigrant origin, with a prevalence of 36.7% for those whose mother lived with a partner and 50.0% for those whose mother did not live with a partner. However, the differences were not significant in either case. For the same cohabitation status, children of immigrant origin showed higher prevalence of OW/OB than natives, which were significant in boys whose mothers were not cohabiting (50.0% vs. 32.1%, *p* = 0.025) and girls whose mothers were cohabiting (44.5% vs. 30.1%, *p* < 0.001).

By age group, the highest prevalence was observed mainly in the 6–9 age group and higher in children of immigrant origin (50.8% vs. 29.6%, *p* = 0.002 in boys and 52.2% vs. 36.2%, *p* = 0.005 in girls).

As for the prevalence of OW/OB as a function of social class, a gradient was detected, namely that the higher the social class, the lower the prevalence of overweight and/or obesity, which was significant in all cases, except in native children. Prevalence ranged from 16.7% (Class I–II) to 46.7% (Class V–VI) in immigrant boys and 28.6% (Class I–II) to 52.3% (Class V–VI) in immigrant girls. Moreover, for the same social class, children of immigrant origin had a higher prevalence of OW/OB, except for Class I–II in boys. These differences were only significant in the cases of Class V–VI, both in boys and girls, corresponding to less qualified working mothers.

The prevalence of OW/OB was significantly higher in children whose mother perceived them to be somewhat heavier or significantly heavier than normal, regardless of the child’s sex or origin. Moreover, these prevalences were higher in girls. Prevalence in this category ranged from 47.9% in native boys to 66.7% in girls of immigrant origin. On the other hand, the prevalence of OW/OB in children whose mother perceived the child’s weight as normal or below normal was significantly higher in children of immigrant origin, both in boys (37.8% vs. 25.2%, *p* = 0.002) and girls (39.8% vs. 26.0%, *p* < 0.001). In addition, the prevalence of OW/OB was significantly higher in children whose mother was overweight/obese, both in children of native and immigrant origin and in boys and girls. Moreover, the prevalence was significantly higher in children of immigrant origin (50.0% vs. 32.9% in boys and 56.4% vs. 36.4% in girls).

Table 3 shows the frequency of consumption of different foods according to the child’s origin and sex. In general, children of immigrant origin had a significantly higher weekly frequency of consumption of fresh fruit, eggs, pasta, rice and potatoes, pulses, and soft drinks with sugar than children of native origin (more than three times per week), and a significantly lower consumption frequency of fish, cold meats, and sausages. When analyzing the results of the Gamma coefficient, significant directionality was detected in the association between the child’s origin and the consumption of the different foods in the case of fresh fruit, soft drinks with sugar and snacks (higher consumption if of immigrant origin in both sexes), cold meats (lower consumption if of immigrant origin in both sexes), eggs, and fish (lower consumption if of immigrant origin in girls), and snacks (higher consumption if of immigrant origin in boys).

Table 4 presents the results of the multivariate analyses. Children of immigrant origin had a higher adjusted risk of OW/OB (OR = 1.67, *p* < 0.001), unhealthy consumption of soft drinks with sugar (OR = 1.38, *p* = 0.014), low physical exercise (OR = 1.65, *p* < 0.001), and use of screens during the week (OR = 1.44, *p* < 0.028). A significant interaction was detected between the child’s origin and educational level on the risk of fast-food consumption and on the risk of screen-use at the weekend. Thus, children of immigrant origin whose mothers had a higher level of education had a higher risk of fast-food consumption than those of native origin (OR = 1.24, *p* = 0.024 and OR = 1.20, *p* = 0.011 in higher vocational training and university studies, respectively), and a lower risk than native children when the mothers had basic–intermediate studies (OR = 0.52, *p* = 0.022). On the other hand, children of immigrant origin whose mothers had basic–intermediate studies had a higher risk of using screens on weekends than those of native origin (OR = 5.23, *p* = 0.001), a lower risk for those with mothers with higher vocational training studies (OR = 0.52, *p* < 0.001), and similar risks for those with mothers with university studies (OR = 1.00, *p* > 0.05).

Girls had a significantly higher risk of unhealthy consumption of sweets (OR = 1.56, *p* = 0.030) and low physical exercise (OR = 2.24, *p* < 0.001), and a lower risk of consumption of soft drinks with sugar (OR = 0.74, *p* = 0.008) than boys.

A gradient was observed that the lower the social class level the higher the risk of OW/OB compared to those belonging to Class I–II (OR = 2.09 for Class V–VI; OR = 1.42 for Class III–IV, *p* < 0.001), as well as a higher risk of low physical exercise (OR = 1.94 for Class V–VI; OR = 1.54 for Class III–IV, *p* = 0.005). A difference was also observed in the consumption of snacks and soft drinks with sugar in children according to social class. It was higher in Class V–VI (OR = 1.279, *p* = 0.044 and OR = 1.285, *p* = 0.003 for Class V–VI vs. Class I–II, respectively).

Finally, it was observed that the older the age, the lower the risk of OW/OB (OR = 0.95, *p* = 0.001) and low physical exercise (OR = 0.88, *p* < 0.001), but the higher the risk of the unhealthy consumption of soft drinks with sugar (OR = 1.14, *p* < 0.001), fast food (OR = 1.13, *p* < 0.001), and use of screens during the week (OR = 1.16, *p* < 0.001) and at weekends (OR = 1.18, *p* < 0.001).

## 4. Discussion

In the present study, it was observed that children of immigrant origin showed higher levels of OW/OB than natives, and a higher adjusted risk of having OW/OB. This is consistent with other studies conducted in the United States [22], Europe [23,24], and Spain [25,26,27,28] which suggest that the immigrant origin of parents may be a risk factor in the development of excess weight in childhood. In Spain, there are several national studies on diet and childhood obesity [29,30], but they do not consider possible differences between natives and immigrants.

Depending on the different socio-demographic characteristics, immigrant-origin children showed higher levels of OW/OB than natives in the case of the mother’s level of education (except in basic–intermediate education, which was higher in natives), in cohabitation status (generally higher in those who do not live with a partner, both in natives and immigrants), and according to social class (the lower social class, the higher the levels of OW/OB in both cases). It is worth highlighting the importance of the mother’s level of education in the development of OW/OB in children, since, although this gradient is not observed at all ages, it is significant to note that the highest prevalence is among children aged 6–9 years. This is relevant because it is at an early age that eating habits are developed and established, and therefore the influence of family eating behavior is a determining factor [31,32]. In the case of families of immigrant origin, culture is a key factor in the preparation and consumption of different dishes and foods, which can also be affected by the process of acculturation. The choice of food type may also be determined by the availability and affordability of food in the family’s immediate food environment [33,34]. Additionally, in different studies [22,23,24,25], socioeconomic status has been associated with the development of excess weight, with a higher prevalence of obesity being found in those with lower incomes, demonstrating data similar to those of the present study. Similarly, the present study shows that, for the same social class, overweight/obesity tends to be higher in children of immigrant origin. This could also be linked to the problem of the access and availability of healthy foods, as they are often more expensive [33].

On the other hand, OW/OB in children in general is also higher when the mother perceives her weight as higher or significantly higher than normal, i.e., when there is actual overweight/obesity. This is an indicator that both perceive the reality as it is, so it would be useful to consider this fact when developing strategies to reduce OW/OB in children. However, it is also observed that in the case of mothers of immigrant origin, they perceived the children’s weight as normal or below normal, but in reality, there was overweight/obesity. In these situations, one explanation could be that it is related to culture and the normalization and acceptance of being overweight as healthy [35], even though physiologically it is not. In previous studies, it has been observed that a high number of parents of overweight or obese children had an inaccurate perception of their children’s weight status [35,36,37]. It would be necessary to go deeper into this aspect and to know how parents perceive the weight status of their children in order to address the problem of OW/OB in these children through awareness-raising and training activities.

As for the relationship between the mother’s and the child’s excess weight, it was observed that when the mother was OW/OB, the children were also OW/OB in both cases. This may be linked to the fact that the children’s diet is strongly influenced by that of the parents, and by the food environment present in the home. Related to this, previous research has shown that OW/OB mothers appear to engage in generally less-healthy feeding practices with their children than mothers of a healthy weight [38]. It is therefore very important to pass on healthy eating habits from parents to children, especially in childhood, as it is what is established during this period that will have a major impact throughout life. In this sense, it would be interesting to carry out activities aimed specifically at parents to change their eating habits. It should also be noted that, although it occurs in both immigrant and native populations, the prevalence of OW/OB is even higher in children of immigrant origin, making it a priority group for intervention.

In relation to the frequency of food consumption, it was observed that children of immigrant origin consumed more fruit, eggs, pasta, rice and potatoes, pulses, and sugary drinks than natives, and smaller amounts of fish, sausages, and cold meats. Food consumption patterns vary between countries, contexts, and cultures, and therefore migration may lead to different eating habits from natives [39,40]. Previous research suggests that even the pattern and rate of dietary change may differ from country to country, and that migrants belonging to the same ethnic group may be at different levels of the acculturation process [41,42,43]. In this sense, there will be foods consumed frequently and traditionally in Spain, such as sausages and cold meats, which in other cultures and countries are not considered a common food. In other countries such as Portugal, it was observed that children of immigrant origin consumed more eggs, chips, fast food, sweets, and sugary drinks [44], which is similar to the results obtained in the present study. In addition, a study conducted by UNICEF in different countries around the world showed that children of immigrant origin are more likely to have an unhealthy diet, characterized by the consumption of sweets and sugary drinks [45].

In addition, it has been observed that children of immigrant origin were at higher risk of consuming sugary soft drinks, exercising less, and using screens more during the week and at weekends. In agreement with these results, a study conducted in adolescents in Spain [46] showed that immigrant children watched more television than native children, obtaining a similar result to the present study. In relation to physical activity, different studies have found that immigrants were less likely to be physically active than natives [44,47,48], as was the case in our study.

Some limitations and strengths should be referred to. Firstly, in the present study the origin of the child was determined on the basis of the mother’s country of birth. It was not possible, due to the design of the survey (this information is only collected for the selected adult), to obtain information on the country of birth of both parents and, therefore, to analyze possible differences. However, the data were also analyzed when the selected adult was the father, and very similar results were obtained. On the other hand, although variables such as breastfeeding, among others, were initially considered for the analysis, they were not included due to their low response. Another issue to bear in mind when assessing excess weight in children is that there are different criteria for assessing BMI in children, and depending on the one used, the interpretation and comparison will vary. In this study, we chose to use the IOTF criterion for the purposes of comparability with other recent studies that have used this same criterion. Furthermore, it should be mentioned that in this study the anthropometric data are self-reported and may differ from the measured data, as is the case in other studies [49,50]. However, given the impossibility of the direct measurement of weight and height in this type of population-based survey, it is common to use self-reported measurements, as there is good agreement between reported and measured data [51]. Finally, given the cross-sectional design of the study, cause–effect relationships cannot be established. Despite the limitations mentioned above, the study has strengths in that it has been conducted on the basis of a national survey and database, with a representative sample.

## 5. Conclusions

Immigrant-origin children have a higher risk of suffering from excess weight and of having different habits that may be determinants of developing OW/OB, such as a higher risk of consuming soft drinks containing sugar, taking little physical exercise, and a greater use of screens. Knowing the socio-demographic variables that may promote childhood obesity, including the child’s origin, would provide an appropriate tool to intervene in the prevention and/or reduction of excess weight in the most vulnerable communities, adapting strategies and policies to each population group and their needs. In this sense, interventions aimed at achieving the acquisition and development of healthy eating habits and patterns, both for children and their families, will have a positive impact on children’s health and on the health of future adults. Therefore, public health policies and interventions aimed at the prevention and treatment of childhood obesity should incorporate a sensitive and clear focus on social and economic inequalities, with an emphasis on the most at-risk groups.

## Figures and Tables

**Table 1:** Average BMIs disaggregated by sex and age of child and country of birth of mother.

BMI								
Country of Birth of Mother		Spain			Other			
Sex	Age	*N*	Mean	Standard Deviation	*N*	Mean	Standard Deviation	Sig
**Male**	2	32	19.03	6.04	12	15.95	1.43	
	3	34	18.08	3.82	12	17.49	6.46	
	4	59	15.84	2.65	19	20.54	5.01	
	5	53	16.90	4.83	11	19.92	6.37	
	2–5	179	17.16	4.44	54	18.73	5.38	0.055
	6	50	19.00	9.57	15	19.90	5.63	
	7	55	16.40	3.58	15	22.68	14.45	
	8	51	18.11	3.20	20	18.48	4.34	
	9	83	17.99	3.84	11	18.59	2.52	
	6–9	239	17.86	5.45	61	19.88	8.12	0.069
	10	36	19.11	3.37	20	19.18	4.81	
	11	35	19.12	3.94	13	20.03	4.24	
	12	45	19.30	3.32	15	21.29	2.76	
	13	39	20.23	3.50	15	20.76	3.50	
	14	55	20.72	3.70	16	19.83	3.21	
	10–14	210	19.78	3.60	80	20.15	3.82	0.442
	Total	628	18.30	4.73	195	19.67	5.89	0.003
**Female**	2	67	16.88	3.81	14	17.84	6.56	
	3	66	16.47	4.08	12	17.10	3.73	
	4	86	18.31	10.13	15	18.77	7.23	
	5	68	16.79	4.44	18	16.20	2.78	
	2–5	287	17.02	5.73	60	18.29	5.33	0.116
	6	91	16.41	2.71	24	18.85	6.78	
	7	96	17.52	3.92	27	17.02	4.44	
	8	103	16.85	3.17	12	19.36	2.39	
	9	101	18.04	3.83	26	19.50	3.33	
	6–9	392	17.23	3.52	89	18.71	4.85	0.008
	10	71	18.21	3.27	16	19.41	4.17	
	11	81	19.19	3.16	14	20.81	5.11	
	12	89	19.74	3.18	10	21.70	5.14	
	13	68	19.88	3.16	10	20.80	3.24	
	14	72	20.05	3.10	11	21.67	2.58	
	10–14	382	19.37	3.16	61	21.11	4.11	0.002
	Total	1061	17.94	4.26	210	19.29	4.91	<0.001

BMI: body mass index.

**Table 2:** Prevalence of overweight/obesity according to socio-demographic characteristics of mothers disaggregated by sex of the child and country of birth of mother.

	Male									Female								
Country of Birth of Mother	Spain				Other					Spain				Other				
	Total	OB/OW			Total	OB/OW				Total	OB/OW			Total	OB/OW			
	*n*	*n*	%	Sig ^(a)^	*n*	*n*	%	Sig ^(a)^	Sig ^(b)^	*n*	*n*	%	Sig b ^(a)^	*n*	*n*	%	Sig ^(a)^	Sig ^(b)^
**Total**	628	185	29.5		195	79	40.5		0.004	1061	321	30.3		210	94	44.8		<0.001
**Educational Level**																		
Basic–Intermediate	45	23	51.1	<0.001	32	10	31.3	0.191	0.083	54	17	31.5	0.187	35	19	54.3	0.394	0.032
Higher vocational training	133	51	38.3		41	20	48.8		0.235	256	95	37.1		30	16	53.3		0.084
University	221	54	24.4		83	41	49.4		<0.001	368	111	30.2		100	43	43.0		0.015
**Cohabitation**																		
Couples	467	133	28.5	0.404	139	51	36.7	0.100	0.065	877	264	30.1	0.736	164	73	44.5	0.797	<0.001
Other	140	45	32.1		50	25	50.0		0.025	172	54	31.4		45	21	46.7		0.055
**Age Group**																		
2–5	179	56	31.3	0.729	54	19	35.2	0.140	0.591	287	90	31.3		60	27	45.0	0.062	0.040
6–9	239	71	29.7		61	31	50.8		0.002	392	142	36.2		89	47	52.8		0.005
10–14	210	58	27.6		80	29	36.3		0.152	382	89	23.3		61	20	32.8		0.110
**Social Class**																		
Class I–II	164	42	25.6	0.415	24	4	16.7	0.023	0.341	290	71	24.5	0.002	21	6	28.6	0.009	0.675
Class III–IV	225	66	29.3		43	17	39.5		0.185	367	102	27.8		52	16	30.8		0.655
Class V–VI	233	74	31.8		122	57	46.7		0.006	399	145	36.3		132	69	52.3		0.001
**Child’s Weight Perception**																		
Normal or below normal	511	129	25.2	<0.001	156	59	37.8	0.138	0.002	885	230	26.0	<0.001	171	68	39.8	0.002	<0.001
Fairly or somewhat above normal	117	56	47.9		35	18	51.4		0.711	176	91	51.7		39	26	66.7		0.090
**Mother’s Overweight/Obesity**																		
Normal	411	113	27.5	0.165	97	34	35.1	0.040	0.140	689	185	26.9	0.001	108	36	33.3	0.001	0.162
Overweight/obesity	210	69	32.9		88	44	50.0		0.005	354	129	36.4		101	57	56.4		<0.001

(a). Differences between the categories of the variable. (b). differences according to the mother’s country of birth.

**Table 3:** Frequency of food consumption disaggregated by sex of child and country of birth of mother.

Country of Birth of Mother		Spain						Other								
Frequency of Weekly Consumption		<1		1–2		≥3		<1		1–2		≥3		Sig ^(a)^	Gamma	Sig ^(b)^
**Fresh Fruit (Excluding Juices)**																
	Male	48	6.80%	139	19.80%	515	73.40%	7	3.10%	36	15.80%	185	81.10%	0.03	0.222	0.007
	Female	57	4.90%	219	18.80%	887	76.30%	9	3.50%	31	12.00%	219	84.60%	0.014	0.248	0.002
	Total	105	5.60%	358	19.20%	1402	75.20%	16	3.30%	67	13.80%	404	83.00%	0.001	0.228	<0.001
	Male *	287	41.70%	349	50.70%	53	7.70%	83	37.20%	119	53.40%	21	9.40%	0.433	0.09	0.199
	Female *	455	39.90%	594	52.20%	90	7.90%	82	32.30%	154	60.60%	18	7.10%	0.047	0.12	0.056
	Total *	742	40.60%	943	51.60%	143	7.80%	165	34.60%	273	57.20%	39	8.20%	0.055	0.103	0.027
**Meat (Chicken, Beef, Pork, and Lamb, etc.)**																
	Male	7	1.00%	421	60.00%	274	39.00%	3	1.30%	120	53.30%	102	45.30%	0.209	0.12	0.117
	Female	11	0.90%	738	63.50%	413	35.50%	9	3.50%	166	64.10%	84	32.40%	0.006	−0.099	0.162
	Total	18	1.00%	1159	62.20%	687	36.90%	12	2.50%	286	59.10%	186	38.40%	0.021	0.012	0.813
**Eggs**																
	Male	66	9.40%	604	86.00%	32	4.60%	22	9.60%	184	80.70%	22	9.60%	0.016	0.15	0.144
	Female	71	6.10%	1019	87.70%	72	6.20%	35	13.50%	205	79.20%	19	7.30%	<0.001	−0.200	0.036
	Total	137	7.30%	1623	87.10%	104	5.60%	57	11.70%	389	79.90%	41	8.40%	<0.001	−0.046	0.51
**Fish**																
	Male	70	10.00%	570	81.10%	63	9.00%	24	10.50%	179	78.20%	26	11.40%	0.531	0.049	0.595
	Female	99	8.50%	949	81.70%	114	9.80%	45	17.40%	204	78.80%	10	3.90%	<0.001	−0.397	<0.001
	Total	169	9.10%	1519	81.40%	177	9.50%	69	14.10%	383	78.50%	36	7.40%	0.002	−0.192	0.002
**Pasta, Rice, and Potatoes**																
	Male	2	0.30%	425	60.50%	275	39.20%	0	0.00%	102	44.70%	126	55.30%	<0.001	0.316	<0.001
	Female	8	0.70%	710	61.20%	442	38.10%	2	0.80%	90	34.60%	168	64.60%	<0.001	0.485	<0.001
	Total	10	0.50%	1135	61.00%	717	38.50%	2	0.40%	192	39.30%	294	60.20%	<0.001	0.413	<0.001
**Bread, Cereals**																
	Male	9	1.30%	33	4.70%	660	94.00%	2	0.90%	8	3.50%	219	95.60%	0.649	0.163	0.318
	Female	9	0.80%	67	5.80%	1086	93.50%	0	0.00%	14	5.40%	244	94.60%	0.355	0.102	0.464
	Total	18	1.00%	100	5.40%	1746	93.70%	2	0.40%	22	4.50%	463	95.10%	0.365	0.133	0.207
**Vegetables, Salads, and Greens**																
	Male	66	9.40%	267	38.00%	369	52.60%	19	8.30%	83	36.40%	126	55.30%	0.751	0.052	0.449
	Female	62	5.30%	439	37.80%	660	56.80%	16	6.20%	80	31.00%	162	62.80%	0.12	0.101	0.128
	Total	128	6.90%	706	37.90%	1029	55.20%	35	7.20%	163	33.50%	288	59.30%	0.207	0.066	0.166
**Legumes**																
	Male	67	9.50%	611	87.00%	24	3.40%	24	10.50%	187	81.70%	18	7.90%	0.016	0.113	0.287
	Female	88	7.60%	1031	88.80%	42	3.60%	34	13.10%	214	82.60%	11	4.20%	0.013	−0.187	0.065
	Total	155	8.30%	1642	88.10%	66	3.50%	58	11.90%	401	82.20%	29	5.90%	0.002	−0.041	0.581
**Sausages and Cold Meats**																
	Male	69	9.80%	292	41.70%	340	48.50%	82	36.10%	63	27.80%	82	36.10%	<0.001	−0.367	<0.001
	Female	121	10.40%	548	47.20%	491	42.30%	77	30.20%	102	40.00%	76	29.80%	<0.001	−0.346	<0.001
	Total	190	10.20%	840	45.10%	831	44.70%	159	33.00%	165	34.20%	158	32.80%	<0.001	−0.350	<0.001
**Dairy Products (Milk, Cheese, and Yogurt)**																
	Male	4	0.60%	15	2.10%	682	97.30%	4	1.80%	4	1.80%	220	96.50%	0.23	−0.136	0.547
	Female	25	2.20%	30	2.60%	1106	95.30%	4	1.50%	12	4.60%	243	93.80%	0.179	−0.132	0.392
	Total	29	1.60%	45	2.40%	1788	96.00%	8	1.60%	16	3.30%	463	95.10%	0.555	−0.110	0.379
**Sweets (Biscuits, Pastries, Sweets, and Jams, etc.)**																
	Male	72	10.30%	205	29.20%	424	60.50%	23	10.10%	54	23.70%	151	66.20%	0.243	0.101	0.164
	Female	71	6.10%	415	35.80%	674	58.10%	21	8.10%	90	34.70%	148	57.10%	0.5	−0.032	0.622
	Total	143	7.70%	620	33.30%	1098	59.00%	44	9.00%	144	29.60%	299	61.40%	0.232	0.032	0.513
**Soft Drinks with Sugar**																
	Male	487	69.50%	164	23.40%	50	7.10%	117	51.30%	80	35.10%	31	13.60%	<0.001	0.341	<0.001
	Female	840	72.70%	230	19.90%	86	7.40%	167	65.00%	66	25.70%	24	9.30%	0.048	0.162	0.021
	Total	1327	71.50%	394	21.20%	136	7.30%	284	58.60%	146	30.10%	55	11.30%	<0.001	0.261	<0.001
**Fast Food (Fried Chicken, Sandwiches, Pizzas, or Hamburgers)**																
	Male	341	48.70%	316	45.10%	43	6.10%	95	41.90%	124	54.60%	8	3.50%	0.029	0.093	0.182
	Female	555	47.90%	543	46.90%	61	5.30%	138	53.70%	107	41.60%	12	4.70%	0.241	−0.107	0.099
	Total	896	48.20%	859	46.20%	104	5.60%	233	48.10%	231	47.70%	20	4.10%	0.418	−0.009	0.853
**Snacks or savoury finger foods (potato crisps, croutons or pickles)**																
	Male	352	50.30%	314	44.90%	34	4.90%	93	40.80%	120	52.60%	15	6.60%	0.04	0.178	0.011
	Female	590	51.00%	504	43.60%	63	5.40%	133	51.80%	111	43.20%	13	5.10%	0.957	−0.017	0.797
	Total	942	50.70%	818	44.00%	97	5.20%	226	46.60%	231	47.60%	28	5.80%	0.268	0.076	0.107
**Natural Fruit or Vegetable Juice**																
	Male	339	48.40%	209	29.80%	153	21.80%	89	39.00%	86	37.70%	53	23.20%	0.034	0.124	0.046
	Female	554	47.80%	365	31.50%	239	20.60%	101	39.00%	105	40.50%	53	20.50%	0.012	0.108	0.053
	Total	893	48.00%	574	30.90%	392	21.10%	190	39.00%	191	39.20%	106	21.80%	0.001	0.116	0.005

* Frequency of daily consumption (a) Significance of chi-square test (b) Gamma significance test.

**Table 4:** Adjusted odds ratios for the risk of unhealthy habits of the child adjusted for sex and age of the child and socio-demographic characteristics of the mother.

Variable			Consumption of Food One or More Times a Week								Physical Exercise		Screen-Use			
	Overweight/Obesity		Sweets		Sugary Soft Drinks		Fast Food		Snacks		No/Some Exercise		More than 1 h/day Screens on Weekdays		More than 1 h/day Weekend Screens	
	OR (CI 95%)	Sig	OR (CI 95%)	Sig	OR (CI 95%)	Sig	OR (CI 95%)	Sig	OR (CI 95%)	Sig	OR (CI 95%)	Sig	OR (CI 95%)	Sig	OR (CI 95%)	Sig
**Sex (Child)**																
Female	1.05 (0.83;1.32)	0.704	1.56 (1.04;2.33)	0.03	0.74 (0.60;0.92)	0.008	0.93 (0.75;1.15)	0.496	0.84 (0.68;1.03)	0.091	2.24 (1.80;2.79)	<0.001	0.87 (0.67;1.14)	0.308	1.25 (0.93;1.68)	0.141
Male	1		1		1		1		1		1		1		1	
**Country of Birth (Mother)**																
Other	1.67 (1.27;2.18)	<0.001	0.70 (0.45;1.11)	0.128	1.38 (1.07;1.79)	0.014	0.52 (0.29;0.91)	0.022	1.05 (0.82;1.34)	0.718	1.65 (1.28;2.14)	<0.001	1.44 (1.04;1.99)	0.028	5.23 (2.02;13.55)	0.001
Spain	1		1		1		1		1		1		1		1	
**Cohabitation**																
Other	0.93 (0.70;1.24)	0.631	1.44 (0.84;2.48)	0.185	1.01 (0.77;1.32)	0.944	1.17 (0.91;1.51)	0.231	0.85 (0.66;1.09)	0.204	1.16 (0.89;1.51)	0.259	0.73 (0.54;1.00)	0.051	0.79 (0.55;1.12)	0.183
Couples	1		1		1		1		1		1		1		1	
**Level of Studies**		0.069		0.355		0.094		0.006		0.258		0.001		0.277		
University	0.86 (0.59;1.24)	0.409	1.46 (0.83;2.57)	0.185	0.73 (0.52;1.02)	0.066	0.81 (0.53;1.23)	0.318	0.76 (0.55;1.06)	0.103	0.56 (0.40;0.79)	0.001	1.26 (0.84;1.89)	0.273	2.19 (1.28;3.74)	0.004
Higher vocational training	1.15 (0.78;1.70)	0.468	1.51 (0.82;2.77)	0.189	0.90 (0.63;1.28)	0.553	1.16 (0.75;1.79)	0.493	0.79 (0.56;1.11)	0.166	0.76 (0.53;1.09)	0.142	1.42 (0.92;2.19)	0.111	2.88 (1.65;5.05)	<0.001
Basic–Intermediate	1		1		1		1		1		1		1		1	
**Social Class**		<0.001		0.784		0.003				0.044		0.005		0.686		
Class V–VI	2.09 (1.29;3.41)	0.003	1.30 (0.62;2.69)	0.486	1.28 (0.84;1.97)	0.252	0.81 (0.54;1.21)	0.3	1.28 (0.87;1.89)	0.215	1.94 (1.27;2.96)	0.002	1.14 (0.70;1.86)	0.608	1.11 (0.62;1.97)	0.724
Class III–IV	1.42 (0.86;2.33)	0.167	1.25 (0.59;2.63)	0.561	0.83 (0.53;1.29)	0.409	0.73 (0.49;1.09)	0.122	0.96 (0.65;1.43)	0.859	1.54 (1.00;2.37)	0.048	1.01 (0.62;1.65)	0.977	0.90 (0.51;1.60)	0.727
Class I–II	1		1		1		1		1		1		1		1	
**Age (Child)**	0.95 (0.92;0.98)	<0.001	0.98 (0.92;1.04)	0.45	1.14 (1.10;1.18)	<0.001	1.10 (1.06;1.13)	<0.001	1.03 (1.00;1.06)	0.082	0.88 (0.85;0.91)	<0.001	1.12 (1.08;1.16)	<0.001	1.18 (1.13;1.24)	<0.001
**Country of Birth x Level of Studies (Interaction)**								0.029								<0.001
Other x University							2.33 (1.21;4.47)	0.011							0.19 (0.07;0.55)	0.002
Other x Higher vocational training							2.40 (1.12;5.13)	0.024							0.10 (0.03;0.31)	<0.001
